# Decision Variants for the Automatic Determination of Optimal Feature Subset in RF-RFE

**DOI:** 10.3390/genes9060301

**Published:** 2018-06-15

**Authors:** Qi Chen, Zhaopeng Meng, Xinyi Liu, Qianguo Jin, Ran Su

**Affiliations:** 1 School of Computer Software, Tianjin University, Tianjin 300350, China; joannaxiaoqi@163.com (Q.C.); mengzp@tju.edu.cn (Z.M.); xinyiliu@tju.edu.cn (X.L.); qgking@tju.edu.cn (Q.J.); 2 The Military Transportation Command Department, Army Military Transportation University, Tianjin 300361, China; 3 Tianjin University of Traditional Chinese Medicine, Tianjin 300193, China; 4 State Key Laboratory of Medicinal Chemical Biology, Nankai University, Tianjin 300074, China

**Keywords:** feature selection, RFE, decision variant, random forest, voting

## Abstract

Feature selection, which identifies a set of most informative features from the original feature space, has been widely used to simplify the predictor. Recursive feature elimination (RFE), as one of the most popular feature selection approaches, is effective in data dimension reduction and efficiency increase. A ranking of features, as well as candidate subsets with the corresponding accuracy, is produced through RFE. The subset with highest accuracy (HA) or a preset number of features (PreNum) are often used as the final subset. However, this may lead to a large number of features being selected, or if there is no prior knowledge about this preset number, it is often ambiguous and subjective regarding final subset selection. A proper decision variant is in high demand to automatically determine the optimal subset. In this study, we conduct pioneering work to explore the decision variant after obtaining a list of candidate subsets from RFE. We provide a detailed analysis and comparison of several decision variants to automatically select the optimal feature subset. Random forest (RF)-recursive feature elimination (RF-RFE) algorithm and a voting strategy are introduced. We validated the variants on two totally different molecular biology datasets, one for a toxicogenomic study and the other one for protein sequence analysis. The study provides an automated way to determine the optimal feature subset when using RF-RFE.

## 1. Introduction

Feature selection is a frequently used technology in the fields of machine learning and statistics, aiming to reduce the high-dimensional feature space by selecting relevant features and removing redundant features. Over the past few years, driven by the applications in a wide range of fields, such as genetics, chemometrics, business etc., feature selection, as one of the most important research areas in high dimensional data analysis, has attracted more and more attention. It can simplify the model and reduce the computational cost to a large extent [1,2]. Compared with feature reduction method, such as principal component analysis, feature selection techniques do not alter the original representations, but merely select a certain number of features which are most informative for classification/regression [3]. Therefore, feature selection is helpful to understand the relationship of features and reveal the inner mechanisms in certain area. For instance, in Su et al.’s study, using a feature selection technique, they found out that fluorescent marker plays an extremely important role in kidney toxicity [4]. In Saeys et al.’s study, they discussed the applications of feature selection techniques in sequence analysis, microarray analysis, and mass spectra analysis, and obtained significant biology mechanism through feature selection [5].

Recursive feature elimination (RFE), is commonly used for feature selection. A ranking of features as well as candidate subsets, with the corresponding accuracy, is produced through RFE. The subset with highest accuracy (HA) or a preset number of features (PreNum) are often used as the final subset. However, this may lead to a large number of features being selected, or with no prior knowledge about this preset number and no human intervention, it is often ambiguous and subjective regarding final subset selection. We call it “decision variant”, which determines the final subset from the group of subsets, and accuracy. A proper decision variant is in high demand to automatically determine the optimal subset. A number of RFE-based feature selection algorithms have been developed, however, there has been hardly any exploration with regard to determining the optimal subset after obtaining a group of subsets and corresponding accuracy. In this study, we conduct pioneering work to explore the decision variant after obtaining a list of candidate subsets from RFE. We provide a detailed analysis for automatically selecting the optimal feature subset using various decision variants in random forest (RF)-recursive feature elimination. Comparisons of these variants are also given, which provide researchers objective criteria to select a proper variant when using RFE, and no prior knowledge about the confinement of the subset is given. A voting strategy for subset selection in the cross-validation with various selection variants was introduced and tested here. We tested our method on two public datasets: Open toxicogenomics project-genomics assisted toxicity evaluation system (Open TG-Gates), which comes from a toxicogenomic study [6], and cell penetrating peptides (CPP) site3 [7], which has been used in protein sequence analysis. The paper is organized as follows: We first give a complete literature review in Related Works. In the Methods Section, we introduce our methodology in detail, followed by experimental results. We give conclusions and discussions in the Conclusions Section.

## 2. Related Works

Over the past few years, a number of feature selection algorithms have been proposed, such as exhaustive searching, forward selection, backward elimination etc. They can be roughly divided into three categories: filter methods, wrapper methods, and embedded methods [8,9,10]. Filter is a method that uses an indicator to evaluate the features, ranks the features based on the index values, and picks features that are at the top of the ranking. Compared to the other two methods, it takes the least time. Wrapper evaluates a feature according to the final performance of the model after adding this feature. Filter method and wrapper method can be used together with various algorithms, while the embedded method selects features as part of the model construction process, and is quite closely integrated with the algorithm itself, thus, the feature selection is completed during the training of the model. Among the feature selection algorithms in the literature, RFE is one of the most popular methods. It was introduced by Guyon et al., for the selection of the optimal gene subsets in cancer classification [11], and was later widely used in other fields, such as DNA microarray studies [12,13], toxicity studies [4], image classification studies [14,15], etc.

Recursive Feature Elimination is commonly used together with many classification algorithms (e.g., support vector machine, RF, etc.) to build more efficient classifiers. A ranking of features as well as candidate subsets is produced through RFE. A list of accuracy values corresponding to each subset is also generated through this procedure. A support vector machine (SVM) based on recursive feature elimination (SVM-RFE) selects patterns using SVM’s weights, and has shown its good feature selection ability. It combines the excellent performance of SVM and the advantage of RFE [11]. Yang et al. used SVM-RFE to maximize the classification accuracy of fault detection by selecting the best combination of the variables [16]; Duan et al. used SVM-RFE to select gene in cancer classification [8]. However, SVM-RFE has its intrinsic defects on the application of data analysis, such as the performance on small dataset is better [17]. Random forest is a widely used machine learning model, which was introduced by Breiman [18]. It has some advantages compared with other algorithms. For instance, it is good at handling the high-dimensional data. A ranking of feature importance which represents their classification contribution can be provided. Compared with other methods, RF-RFE has been proven to be more effective, which can use fewer features to get a higher classification accuracy [19]. Granitto et al. used the RF-RFE algorithm to accomplish the feature selection in Proton Transfer Reaction – Mass Spectrometry (PTR-MS) study [17]; In Chen et al.’s study, they proposed an enhanced recursive feature elimination method to classify small training samples [20].

The combination of RFE with classification algorithms leads to a lower data dimension and higher computation efficiency. However, there were problems in terms of selecting the optimal subset rise in the procedure of RFE. Usually, a number *N* to determine how many features are selected is often set in advance. Then, the top *N* features from the ranking are selected as the final subset. If *N* is not known in advance, using what variant to decide the optimal subset is often ambiguous and subjective. Besides a preset number, most studies used the subset corresponding to the HA, or relevant variants to determine the optimal subset.

**Figure 1:**
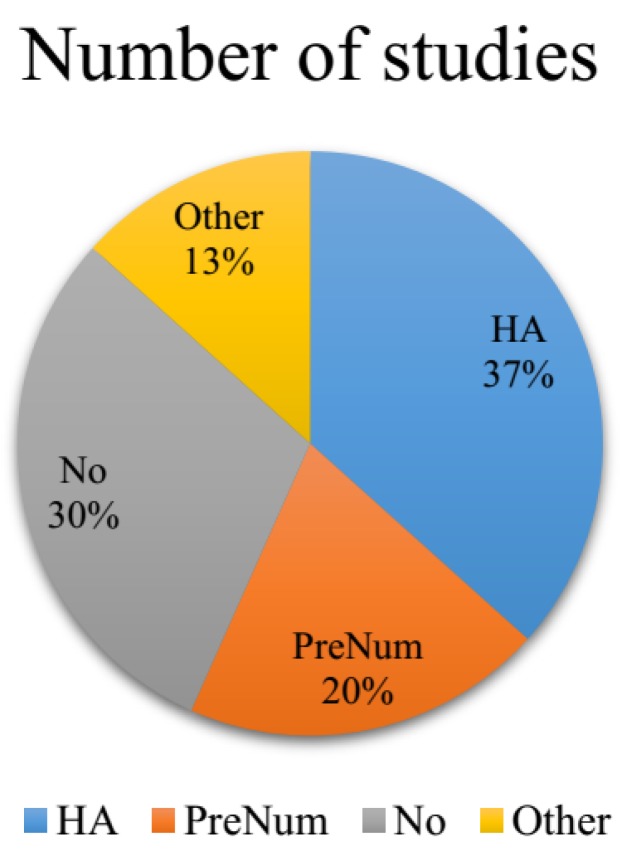
The statistical analysis of 30 most recent publications which used recursive feature elimination (RFE) for feature selection. HA: Used the highest classification accuracy as the decision variant; PreNum: Used a pre-defined number of features as variant; No: Represents that no choice was made; Other: sed other variants for feature selection.

In order to have an overall view of the variants used currently, we analyzed 30 most recent publications which used RFE for classification/regression. A statistics conclusion was given (see Figure 1). In these papers, the features were sorted according to their importance. The least important features were removed, and the features used for classification were updated iteratively. Meanwhile, the classification accuracy of each feature subset was also provided in this procedure.

In these 30 studies, we found that the most commonly used selection variant is the highest accuracy (HA). Of these 30 studies, 11 used HA as selection variant [16,21,22,23,24,25,26,27,28,29]. In this method, the optimal feature subset was determined when the classification accuracy achieves the highest or a certain percentage of the HA, e.g., 90%. For instance, in Yang et al.’s study, five features were selected when the accuracy achieved was the highest [27].

There are six studies which selected the subsets according to a pre-defined number [19,21,30,31,32,33]. In this method, within a certain accuracy scope, the number of selected features is not the same according to different applications. Tiwari et al. selected the top 50 features to compare the classification accuracy, while others might select only less than ten features [32].

Besides these, four studies used other selection variants [34,35,36,37]. Qian et al. used Least Square Support Vector Machine and RFE to select the optimal feature subset [35]. They claimed that comparing with other methods, they could reach the same accuracy using fewer features, which shortened the execution time and increased the computation efficiency. Furthermore, there are nine studies which listed the accuracies or importance but did not make a choice [38,39,40,41,42,43,44,45]. Song listed the classification accuracy using different feature numbers, and drew the curve for analysis, but no choice for optimal feature subset was given [43].

## 3. Methods

A number of RFE-based feature selection algorithms have been developed over the years, however, there is not much available for the optimal feature subset selection after obtaining a group of subsets and corresponding accuracy. We looked at into this issue as a pioneering work. We call it “decision variant”, which determines the final subset from the group of subsets and accuracy. We will introduce this in detail, in this section.

### 3.1. Datasets and Preprocessing

In our study, we used two datasets for model building and evaluation, the first data is TG-Gates_500 and the second is CPPsite3.

TG-Gates_500 is from the Open TG-GATEs database. It stores gene expression profiles and toxicological data, including biochemistry, hematology, and histopathology findings, with pathology imaging from the in vivo studies, and cytotoxicity information from the in vitro studies. Preprocessed human in vitro data containing 500 gene expression profiles of cells of 111 drugs (45 positive and 66 negative) after treatment with compounds was used in our study (to test our methods more efficiently, we randomly picked 500 genes from the whole gene profiles). The preprocessed data was extracted from Toxgates, which is an online tool for Open TG-GATEs analysis [46]. It processed the raw AffymetrixGeneChip data using the Affy package [47] from R. More details about the Open TG-GATEs and the preprocessing operation can be found in [6] and [46], respectively.

CPPsite3 was proposed by Gautam et al. [7] (website, http://crdd.osdd.net/raghava/cellppd/dataset.php), is frequently used to identify the uptake efficiency of CPPs. Cell-penetrating peptides have been successfully applied for the delivery of therapeutic molecules, both in vitro and in vivo. Cellular delivery using CPPs has great potential as therapeutics in gene therapy or cancer treatments. Accurate identification of the uptake efficiency of CPPs is regarded as the prerequisite to an in-depth elucidation of their molecular functions and to reveal their medical applicability. This dataset contains 187 high-uptake efficient CPPs as positives, and the equal number of low-uptake efficient CPPs as negatives. A total of 188 features were extracted for prediction.

To remove the noise and outliers, we conducted some preprocessing operations for the two datasets. The data was normalized to a range [−1, 1] using the formula:

(1) f=2×(f−fminfmax−fmin)−1,

where ***f*** is the feature vector; *f_max_* is the maximum, and *f_min_* is the minimum in ***f***.

### 3.2. Random Forest Classifier

Random forest, which was proposed by Breiman [18], has become one of the most popular classifiers. The RF comprises multiple decision trees, behaving as an ensemble classifier. For the training process, it uses the boot-strap resampling technique to randomly select sample subsets in each decision tree. The final classification result of the RF is determined by the scores derived from all the decision trees. The classification error depends on every tree’s ability of classification, as well as the correlation between the trees.

The RF is a powerful classifier that can perform effectively and efficiently, and has been widely applied in a number of fields [17,30]. As compared with other machine learning algorithms (e.g., support vector machine), the RF has several advantages, such as unbiased estimator, easy to parallel, etc. One key advantage of RF is the importance measure, which reveals the impact of each variable of the predictor. Features with large importance values are ranked as more important than features with small values. The importance provides a method to evaluate the contribution of each feature. It is measured as follows. Firstly, the out-of-bag error is calculated for each decision tree. Then, the values of one feature are permutated across all the test samples, and the out-of-bag error is calculated again. The difference between the two out-of-bag errors measures the importance of that feature. If the error exhibits a large increase, that feature is important.

### 3.3. Feature Selection Using Random Forest—Recursive Feature Elimination 

High-dimensional data often contains a lot of redundant and irrelevant information, which reduces the efficiency of the predictive models for classification [48]. In order to build efficient and effective predictive models, it is, therefore, necessary to select a subset with most discriminative features. In this study, we reduced the dimension of feature space using the RF algorithm, combining it with the RF-RFE. It is supposed that data redundancy is eliminated, and yields more compact feature subsets.

**Figure 2:**
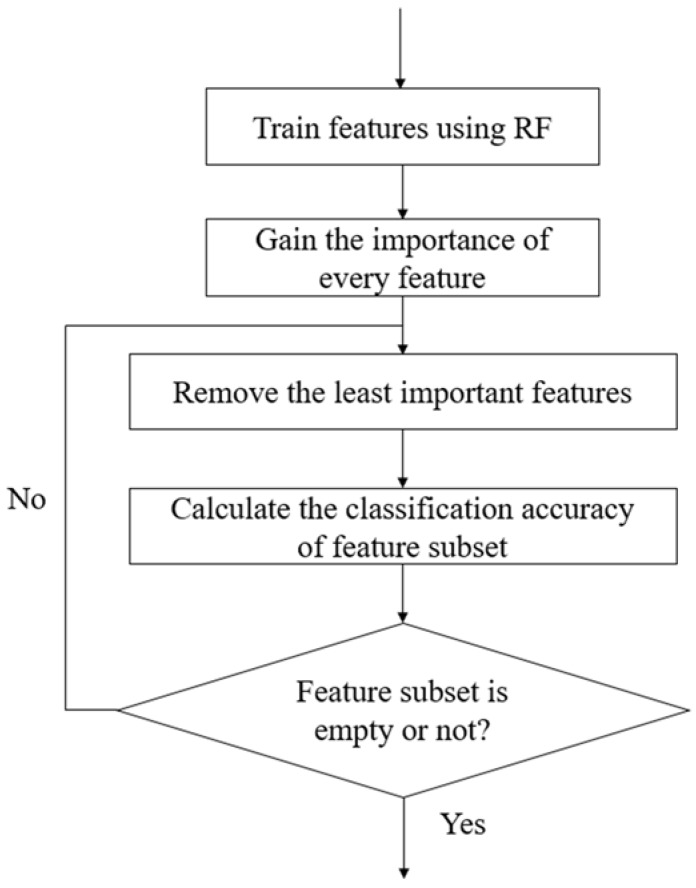
The main procedure of the recursive feature elimination (RFE) method.

The procedure of the RF-RFE method is illustrated in Figure 2. Firstly, we trained our model using the RF algorithm based on the training data, and acquired every feature’s importance according to their classification contribution. Then, the features were sorted from high to low according to their importance. A ranking of features was obtained in this step. Lastly, we eliminated the least important feature, and then used the updated features to re-train the RF model, and obtained the classification performance using the new feature set. This is an iterative procedure until the feature set is empty. After the RF-RFE, a list of performance measurement values corresponding to each subset was produced. Based on the list of values, we explored the decision variant used for subset selection.

### 3.4. Decision Variants for Recursive Feature Elimination

**Figure 3:**
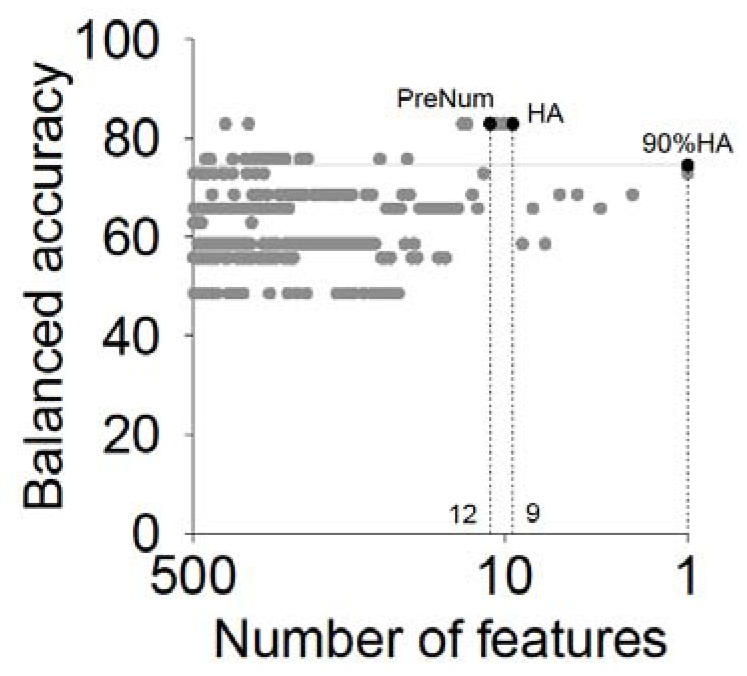
The three variants we analyzed in this study: HA, 90% HA, and PreNum (equals to 12). The result was analyzed based on the TG-Gates_500 data.

The purpose of feature selection is to determine an optimal feature subset that can balance the feature number and classification accuracy at the same time, achieving the goal of dimension reduction and accurate prediction. A feature subset with a good discrimination ability, as well as dimension reduction ability, is the ultimate goal of feature selection. As described earlier, there are mainly two types of variants to determine the optimal feature subset after obtaining a list of accuracies and feature importance from RFE. The first type is the HA or variant related to HA, and the other one is a PreNum. For HA variant, the subset corresponding to the HA (or certain percentage of HA) is selected as the optimal feature subset; For PreNum, top ranked PreNum features sorted by importance are selected as the optimal feature subset. In our study, we explored three variants, HA, 90% of HA (90% HA in short), and PreNum, to decide the number of optimal feature subsets. Analysis, as well as comparisons between the three variants, were provided as follows. Assuming we have, in total, p feature subsets, denoted as *f_sub_* after RFE, the optimal feature subset *F_sf_* using these three variants in each fold is defined as below:

(2) $$F_{sf}(\mathrm{HA})=f_{sub}(\mathrm{HA})|Acc(f_{sub}(\mathrm{HA}))=\arg\max Acc(f_{sub}),$$

(3) $$F_{sf}(90\%\mathrm{HA})=f_{sub}(90\%\mathrm{HA})|Acc(f_{sub}(90\%\mathrm{HA}))=\arg\max90\%\times Acc(f_{sub}),$$

(4) $$F_{sf}(\mathrm{PreNum})=f_{sub}(\mathrm{PreNum})|\mathrm{Feature}\;\mathrm{number}\;\mathrm{in}\;f_{sub}(\mathrm{PreNum})=\mathrm{PreNum},$$

where *Acc*() is the function to calculate the accuracy or balanced accuracy. The illustration of the three variants is shown in Figure 3. In this example, we had 500 features in total. We removed one feature in each iteration, and obtained 500 feature subsets. There are 9, 1, and 12 features corresponding to HA, 90% HA and PreNum respectively, where the HA equals to 82.85%, 90% HA equals to 74.57% and the PreNum was set to 12.

### 3.5. Voting Strategy for Subset Selection after Cross-Validation

**Figure 4:**
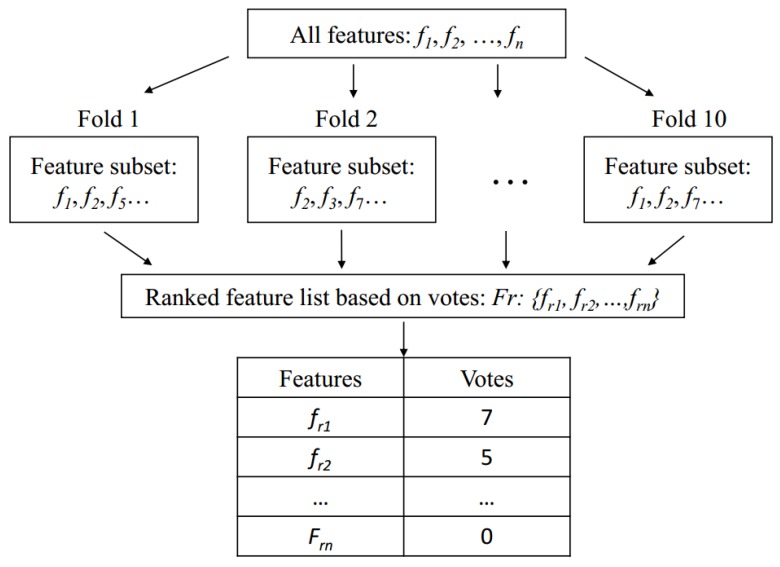
Voting strategy to select the optimal feature subset after the 10-fold cross-validation. Here, we assume that the top two ranked features have votes 7 and 5, respectively.

In our study, a 10-fold cross-validation was used to estimate the performance of the re-trained RF model. We used a voting strategy to determine the final feature subset after the cross-validation. In each fold, one set of features was selected using RFE. Then, ten feature subsets were obtained after the 10-fold cross-validation. We gathered all the selected features in a candidate pool and counted the votes, *v_f_*, across all the folds for each feature. This procedure for the vote calculation is illustrated in Figure 4.

Then, the vote for each feature was used to determine if the feature should be incorporated into the final feature subset. The final selected feature set *F*_s_ with *k* features is obtained as follows:

(5) $$\begin{array}{l} F_{s}=F_{r}:\{f_{r1},...,f_{rk}\}|\max(Acc(F_{r},v_{0})),\\ with\;v_{f}>\nu_{0},\;v_{f},\nu_{0}\in (0,9). \end{array}$$

where *F_r_* is the ranked features according to the votes; *Acc()* calculates the balanced accuracy values; and *F_s_* is the selected subset. As we used ten-fold cross validation, the *υ_f_* ranges from 0to 10 meaning being selected between zero times to ten times. Then, we tested the performance using features whose *υ_f_* is larger than the threshold *υ_0_*. Therefore, *υ_0_* is between 0 and 9. Thus, we have ten combinations of features with votes larger than that when *υ_0_*= 0 to *υ_0_*= 9. We picked the combinations which gave the highest balanced accuracy as the final subset, meanwhile, the *υ_0_* is automatically obtained through this process.

To keep the training and test data independent of each other, we divided the data into four parts. In our feature selection procedure, in each fold, X_1_, X_2_, and X_3_ were used for feature selection. Specifically, X_1_ and X_2_ were used to tune the parameters for each possible subset; then, we used X_1_ plus X_2_ to train an RF and tested on X_3_. Here, from X_3_, we obtained the *Acc*(*f*_sub_), which would be used for optimal subset selection, meanwhile, the importance of the tested feature was obtained. Then, we updated the feature subset using the importance. After looping through all the possible subsets, the complete *Acc*(*f*_sub_) set was used for extracting variants to determine the final subset. Finally, we used the X_4_ based on the selected subset to report the result.

### 3.6. Performance Measurements

In this study, we used 10-fold cross-validation method to evaluate the classification performance [4]. In 10-fold cross-validation, a dataset is randomly partitioned into 10 subsets. Of the 10 subsets, a single subset is retained as the validation data to test the model, and the remaining nine subsets are used as training data. The ten outputs from the ten folds can be averaged (or otherwise combined) to produce the final performance estimation. As described earlier, at the beginning of each cross-validation loop, the datasets were normalized to the same range (−1, 1).

Sensitivity, specificity and balanced accuracy are three important metrics which are commonly used for performance evaluation. The three metrics are formulated as follows:

(6) $$\begin{array}{c} \mathrm{Sensitivity}\;=\;\frac{\mathrm{TP}}{\mathrm{TP}+\mathrm{FN}}\times 100\%,\\ \mathrm{Specificity}=\frac{\mathrm{TN}}{\mathrm{TN}+\mathrm{FP}}\times 100\%,\;\mathrm{and}\\ \mathrm{Balanced}\;\mathrm{accuracy}\;(\mathrm{acc})\;=\frac{\mathrm{Sensitivity}+\mathrm{Specificity}}{2} \end{array}$$

where TP, TN, FP, and FN represent true positive, true negative, false positive, and false negative, respectively.

In our study, we performed all the analysis using the R statistical environment (v3.3.1) on a personal computer equipped with an Intel Core i7-6700K processor and Windows 7 operating system (Microsoft, Redmond, WA, USA). We used “randomForest” library (v4.6-12) under the R environment (https://www.r-project.org/) to perform RF classification. It takes about 30 min to select optimal feature subsets for dataset TG-Gates_500.

## 4. Experimental Results

### 4.1. Performance Using the Voting Strategy

**Figure 5:**
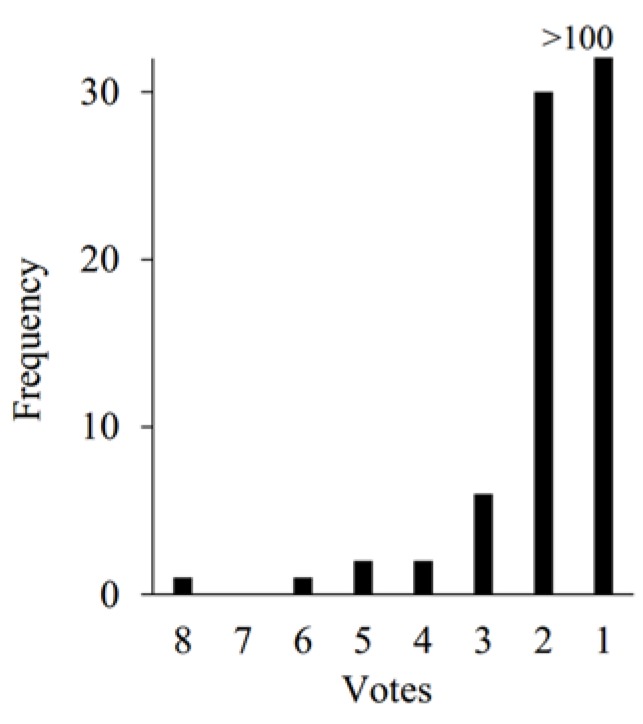
The frequency of votes of the selected features in the candidate feature pool.

We validated the model construction procedure through a 10-fold cross-validation. As described earlier, after the cross-validation, a voting strategy was conducted to determine the final subset. All features selected in each fold were gathered into a candidate feature pool. Features receiving majority votes in the pool were chosen as the candidates in the final subset. Results of TG-Gates_500 data using HA variant were provided here as an example. In Figure 5, a total of 151 genes (features) out of 500 were dropped into the pool. The frequency at which each feature appeared is presented in the figure.

In Figure 5, it is shown that most of the features (109/151) only appear once in the selection results. Merely two features get votes larger than five. The features appearing for a few times indicate their significance in prediction. Classification using such features is supposed to achieve a better result. Therefore, we further conducted experiments to observe the performance using features with votes larger than a threshold *υ*_0_. The performance using different *υ*_0_, as well as no feature selection (without FS), are shown in Table 1. As the largest vote was 8, we set υ_0_ from 0 to 7.

From Table 1, it shows that the RFE with cross-validation improves the performance without any feature selection from 47.57% to 72.78% of balanced accuracy, 13.33% to 62.22% of sensitivity, and 81.82% to 87.88% of specificity. We obtained the highest balanced accuracy using 12 features with votes larger than that *ν*_0_ which is set to 2, indicating the combination of these features is most discriminative and informative for prediction among all the combinations.

### 4.2. Performance Using Different Decision Variants

Following the RFE procedure, we constructed the models, calculated the importance, obtained the subsets and their corresponding performance, and then selected the optimal subset from the accuracy list according to some variants. Here we analyzed and compared three variants HA, 90% HA, and PreNum. The subsets corresponding to HA, 90% HA, and PreNum were selected as the final feature subset. We tested the performance using the selected feature subset, and show their performance for the classification of the TG-Gates_500 data and CPPsite3 in Table 2 and Table 3. The performance without any feature selection is also shown here.

We present the classification results using HA, 90% HA, and PreNum for TG-Gates_500 in Table 2 and CPPsite3 in Table 3. In our study, the PreNum was set according to the number of features selected using HA. From the two tables, compared with the result without any feature selection, it shows that using RF-RFE can greatly improve the performance both for TG-Gates_500 and CPPsite3. The balanced accuracy can be improved from 47.57% to 77.27% for TG-Gates_500 data, and 65.24% to 70.05% for CPPsite3; the feature number can be reduced from 500 to 12 and 188 to 17 for two datasets, respectively. The RF-RFE is effective in performance improvement and model simplification.

Using the RF-RFE method, for the TG-Gates_500 data in Table 2, it can be seen that the number of selected features ranges from 12 to 26, balanced accuracy ranges from 72.78% to 77.27%, sensitivity ranges from 62.22% to 66.67% and specificity ranges from 83.33% to 87.87%. Feature subset selected via 90% HA gives the best performance (77.27%, 66.67%, and 87.87% of balanced accuracy, sensitivity, and specificity, respectively) while HA variant selects the most compact feature subset (12 features were selected) yet has lowest performance (72.78%, 62.22%, and 83.33% in balanced accuracy, sensitivity and specificity respectively) among the three variants. Furthermore, The PreNum is not as good as the other two at dimension reduction with 26 features being selected.

For the CPPsite3 in Table 3, the number of selected features ranges from 17 to 24, balanced accuracy ranges from 68.18% to 70.05%, sensitivity ranges from 64.17% to 67.91% and specificity ranges from 72.19% to 73.26%. Both HA and PreNum achieve the highest balanced accuracy (70.05%). Highest accuracy and 90% HA selected the smallest number features (17 features were selected), while 90% HA’s discrimination ability was not as satisfactory (68.18%) as HA and PreNum. PreNum does not have a good performance at dimension reduction either (24 features were selected).

Overall, from the results above, it shows that the three variants can all achieve a higher accuracy for both datasets compared with the results without any feature selection. However, in terms of feature reduction, HA preserves the least number of features while PreNum keeps largest number of features. Besides, the setting of the PreNum requires some prior knowledge about the data, which may change according to the specific applications.

## 5. Conclusions

Classification accuracy and feature number are the two key indicators for feature selection algorithms. In order to build a precise predictive model, it probably requires sufficient information for model training. Intuitively, this can be done via including as many features as possible, which may reduce the classification efficiency. Nevertheless, if a smaller feature subset is chosen to improve the classification efficiency, the classification accuracy may decrease. Therefore, a good feature selection method should balance both the accuracy and feature number well. Our studies provide a solution for the RFE algorithm. Solutions for RFE inside cross-validation are also considered here.

In this paper, we conduct pioneering work to explore the decision variant after obtaining a list of candidate subsets from RFE. We first give a complete literature review and summary about the decision variants used in the current studies related to RFE. The most commonly used variants are related to the HA and a preset size of feature set. Then, we provide a systematic pipeline to select the optimal feature subset in RF-RFE when cross-validation was also carried out. A voting scheme was used and validated in this study. Secondly, we explored three most commonly used decision variants, the HA, the 90% HA and PreNum, for the selection of the optimal feature subset in RFE. Analysis, as well as comparisons between the three variants was provided. The results using RFE and the results without any feature selection were also shown here. The method was tested on two totally different types of datasets, the TG-Gates_500 for toxicogenomic studies, and CPPsite3 for protein sequence prediction. From the results, firstly, we obtained a rule for the selection of final feature subset outside the cross-validation. Secondly, the results compared with that without any feature selection show that RFE indeed improves the quality of the model, as well as makes the process of model construction more efficient. Furthermore, observing the results of using different variants for prediction, in terms of the performance metrics, we can see that the 90% HA achieves the best for TG-Gates_500, while HA and PreNum achieve the highest balanced accuracy for CPPsite3. In terms of dimension reduction, HA preserves the smallest number of features for both datasets and PreNum obtains the largest number of features. The HA and 90%HA select the features in an automated way, however, the setting of PreNum requires some prior knowledge about the data, which is subjective and may cause bias in the results. However, this may be more flexible for different requirements of applications. Our finding provides criteria for the determination of decision variants, which has potential applications in a wide range of areas using RFE to simplify the computation.

We will test the method and variants on more types of data in the following work, probably on some sequence analysis data or some geographic data. Furthermore, our future works will concentrate on the proposal of a novel decision variant, which should provide an objective criterion for the selection of most informative features and better balance the feature number and classification accuracy.

## Figures and Tables

**Table 1:** The performance using feature with votes larger than a threshold *υ*.

υ	Number of Features	Balanced Accuracy (%)	Sensitivity (%)	Specificity (%)
7	1	52.93	42.22	63.64
6	1	52.93	42.22	63.64
5	2	57.47	42.22	72.73
4	4	61.21	46.67	75.76
3	6	66.41	55.56	77.27
2	12	72.78	62.22	83.33
1	42	66.16	44.44	87.88
0	151	55.40	24.44	86.36
Without FS	500	47.57	13.33	81.82

**Table 2:** Classification performance using three selection variants and performance without any feature selection for TG-Gates_500.

	Number of Features	Balanced Accuracy (%)	Sensitivity (%)	Specificity (%)
HA	12	72.78	62.22	83.33
90% HA	17	77.27	66.67	87.87
PreNum (12)	26	75.40	64.44	86.36
Without FS	500	47.57	13.33	81.82

**Table 3:** Classification performance using three selection variants and performance without any feature selection for CPPsite3.

	Number of Features	Balanced Accuracy (%)	Sensitivity (%)	Specificity (%)
HA	17	70.05	66.84	73.26
90% HA	17	68.18	64.17	72.19
PreNum (17)	24	70.05	67.91	72.19
Without FS	188	65.24	61.50	68.98
